# Retraction: Young plasma reverses anesthesia and surgery-induced cognitive impairment in aged rats by modulating hippocampal synaptic plasticity

**DOI:** 10.3389/fnagi.2023.1349869

**Published:** 2023-12-15

**Authors:** Frontiers Editorial Office

The Publisher retracts the cited article.

Following publication, concerns were raised regarding the integrity of the images in the published figures. Areas of duplication were identified in Figures 7E and 8D.

The authors failed to provide a satisfactory explanation during the investigation, which was conducted in accordance with Frontiers' policies. As a result, the data and conclusions of the article have been deemed unreliable and the article is retracted.

This retraction was approved by the Chief Editors of Frontiers in Aging Neuroscience and the Chief Executive Editor of Frontiers. The authors did not agree to this retraction.

